# Emphasis on peripheral vision is accompanied by pupil dilation

**DOI:** 10.3758/s13423-023-02283-5

**Published:** 2023-04-17

**Authors:** Ana Vilotijević, Sebastiaan Mathôt

**Affiliations:** Department of Psychology, University of Groningen, Grote Kruisstraat 2/1, 9712TS, Groningen, The Netherlands

**Keywords:** Pupil, Vision, Attention

## Abstract

People are best able to detect stimuli in peripheral vision when their pupils are large, and best able to discriminate stimuli in central vision when their pupils are small. However, it is unclear whether our visual system makes use of this by dilating the pupils when attention is directed towards peripheral vision. Therefore, throughout three experiments (*N* = 100), we tested whether pupil size adapts to the “breadth” of attention. We found that pupils dilate with increasing attentional breadth, both when attention is diffusely spread and when attention is directed at specific locations in peripheral vision. Based on our results and others, we propose that cognitively driven pupil dilation is not an epiphenomenal marker of locus coeruleus activity, as is often assumed, but rather is an adaptive response that reflects an emphasis on peripheral vision.

**Supplementary Information:**

The online version contains supplementary material available at 10.3758/s13423-023-02283-5.

## Introduction

Spatial attention is commonly seen as a spotlight that enhances processing for a specific spatial location. Importantly, the size of this spotlight is not constant but adapts to the demands of the situation (Eriksen & St. James, 1986; Eriksen & Yeh, 1985; LaBerge, 1983; Greenwood & Parasuraman, 1999, 2004; Lawrence et al., 2020; Müller et al., 2003). For example, when trying to find a certain figure in a book, your attention is initially broadly distributed as you are flipping through the pages. Yet, once you have found the figure, your attention narrows and focuses on the details of the figure and on the caption underneath. Attentional breadth refers to the size of this spotlight, which gets bigger when attention is diffusely spread across the visual field, thus also encompassing peripheral vision, and smaller when attention is focused centrally, encompassing only (para)foveal vision (Brocher et al., 2018; Mathôt, 2020).

There is an inverse relationship between the breadth of attention and visual processing quality: Broadly distributed attention results in parallel but superficial processing, while narrowly distributed attention allows for detailed scrutiny. These functional differences in processing quality between broadly and narrowly focused attention map onto physiological differences between the foveal and peripheral retina; firstly, the density of cones is highest near the fovea, and declines toward the periphery (Hirsch & Curcio, 1989; Pumphrey, 1948; Rosenholtz, 2016); furthermore, in the fovea, photoreceptors have one-to-one connections to ganglion cells, which enables photoreceptor input to be transmitted with minimal loss of spatial information, while in the peripheral retina many photoreceptors (both rods and cones) project to a single shared ganglion cell, thus losing spatial information. Because of these physiological properties of the retina, foveal vision provides higher visual acuity than peripheral vision does (Curcio et al., 1987; Wolfe et al., 2009).

These well-established physiological differences between peripheral and foveal vision also have implications for the optimal size of the pupil. When the pupil is constricted, only a small part of the lens is exposed, thus lessening optical distortions that cause optical blur (Mathôt, 2018; B. Wang & Ciuffreda, 2006), in turn resulting in high visual acuity and enhancing discrimination performance for foveal stimuli (Mathôt & Ivanov, 2019). In contrast, when the pupil is dilated, a larger part of the lens is exposed, thus allowing more light to enter the eye, in turn resulting in increased visual sensitivity and enhanced detection performance for peripheral stimuli (Mathôt & Ivanov, 2019). The size of the pupil therefore reflects a trade-off between visual acuity and sensitivity, where small pupils favor acuity over sensitivity. Crucially, due to the physiology of the retina, high visual acuity (and thus a small pupil) is mainly beneficial for foveal vision, whereas it is largely wasted on peripheral vision.

The triangular link between pupil size, behavioral performance, and attentional breadth has important implications for the Adaptive-Gain Theory (AGT; Aston-Jones & Cohen, 2005), which posits a link between behavior and pupil size, but only does so descriptively, without explaining *why* this link exists. Specifically, the AGT differentiates between two modes of behavior—*exploitation* and *exploration*—and relates these to locus coeruleus (LC) activity. Information about task utility (how rewarding a task is) would drive two modes of LC activity: phasic and tonic (Aston-Jones & Cohen, 2005; Usher et al., 1999). Phasic activity would reflect on-task behavior (exploitation), during which pupil size is moderate-to-small, while tonic activity would reflect disengagement from the current task in favor of other tasks (exploration), during which pupil size is large.

Here we propose that the increased pupil size as observed during exploration behavior reflects an increased emphasis on peripheral vision; more specifically, as task utility decreases, people become *less* focused on what they are doing, typically a task relying on narrowly focused attention and foveal vision (such as reading), and *more* focused on what they might be doing next, which involves broadly distributed attention and peripheral vision. In other words, we propose that the pupil-size–behavior link as posited by the AGT reflects *sensory tuning*: an optimization of pupil size for the current situation (Mathôt, 2020). However, evidence for this form of sensory tuning is still largely missing. Therefore, here we seek to directly answer the following question: Does pupil size automatically increase as participants attend further into the periphery—that is, with increasing attentional breadth?

Several studies have examined the relationship between attentional breadth and pupil size (Brocher et al., 2018; Daniels et al., 2012; Klatt et al., 2021; Kolnes et al., 2022). Brocher et al. (2018) and Klatt et al. (2021) presented stimuli at varying levels of eccentricity (distance from fixation), with a cue indicating the eccentricity of an upcoming target as a manipulation of attentional breadth. Results showed that pupil size increased with increasing attentional breadth. However, performance was poorer at the far- and medium-eccentricity conditions as compared with the near-eccentricity condition; this makes it impossible to disentangle whether the increase in pupil size simply originated from greater mental effort (because the task was more difficult), or from attentional breadth per se.

Daniels et al. (2012) exposed participants to stimuli in a diamond-like configuration, with a cue directing attention to either the central or peripheral stimuli; for example, in one experiment, the luminance of a central dot indicated either narrow or broad attention. The results again showed that pupil size increased with increasing attentional breadth. However, it was unclear if cues were counterbalanced, and in several experiments visual input differed between narrow- and broad-attention trials, which makes it impossible to separate the effect of attentional breadth from the effect of visual stimulation.

Finally and most recently, Kolnes et al. (2022) manipulated attentional breadth by cueing (with a sound) either a small or a large circle, and asked participants to locate a gap in the cued circle. The results showed that pupil size increased when the larger circle was cued, that is, with increasing attentional breadth. The authors aimed to carefully control for common confounds such as visual input and gaze position, but it still remained unclear if auditory cues were counterbalanced; again this is crucial because not only visual but also auditory stimuli affect pupil size (Gingras et al., 2015). In addition, task difficulty differed between conditions, although in this case the task was easier, rather than more difficult, for the far eccentricity as compared with the near eccentricity (i.e., opposite to the paradigm used by Brocher et al., 2018; Klatt et al., 2021).

**Fig. 1:**
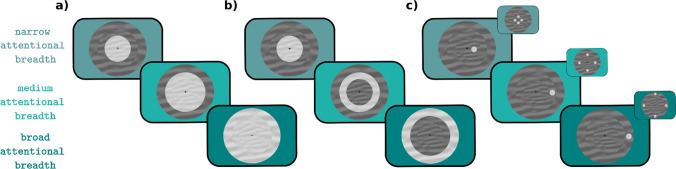
Different operationalizations of attentional breadth. **a)** Attentional spotlight in the shape of a filled circle. **b)** Attentional spotlight in the shape of an annulus*.* **c)** Attentional spotlight directed towards a single location in central or peripheral vision. *Note*: Different background colors represent different levels (narrow, medium, broad) of attentional breadth across all three operationalizations. (Color figure online)

**Fig. 2:**
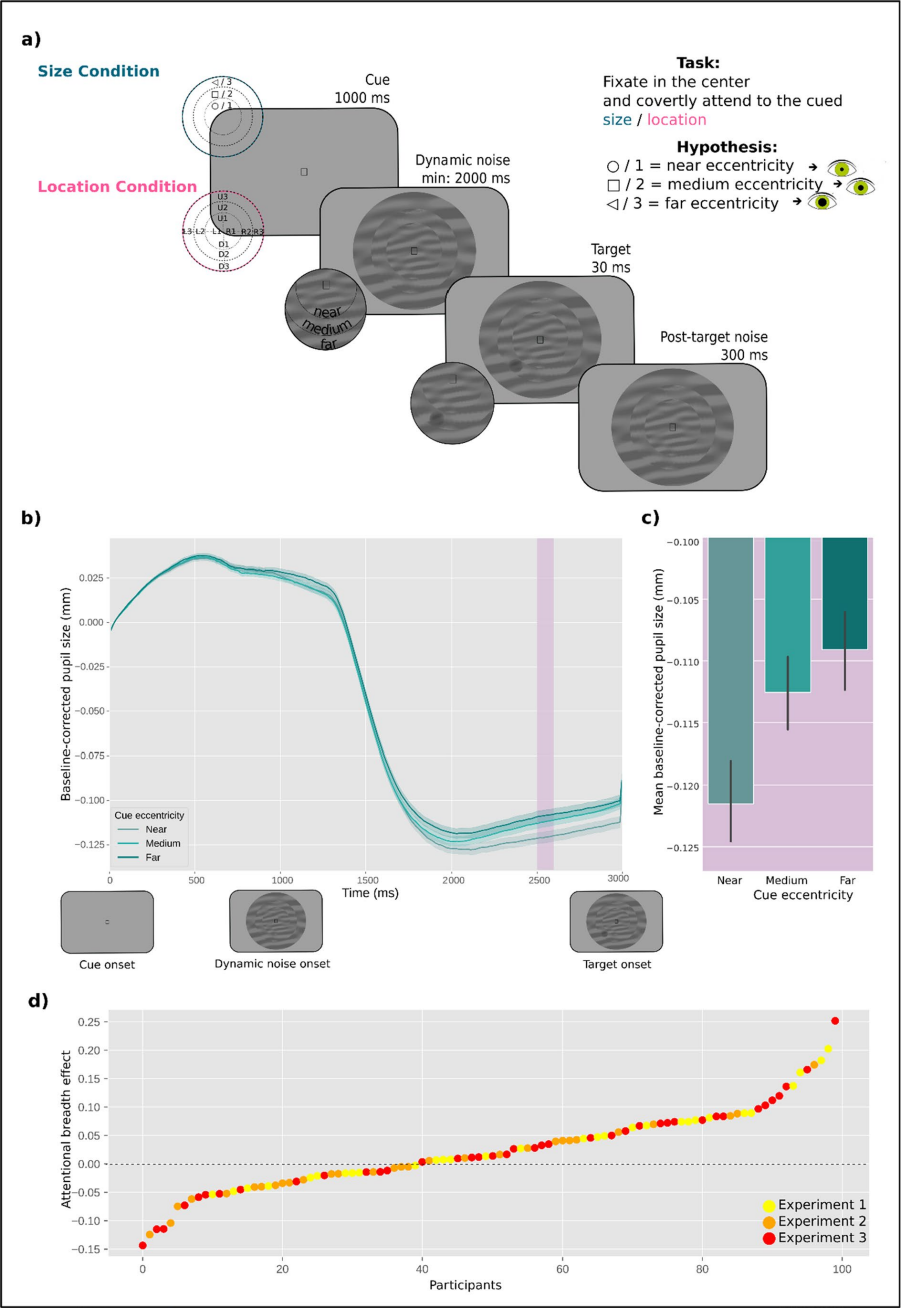
**a)** Schematic trial sequence. In the Size Condition, participants’ attention was cued to one of the differently sized annuli. In the Location Condition, participants' attention was cued to a specific location within one of the annuli. Participants reported whether a target was a luminance increment or, as in this example, a luminance decrement embedded in a dynamic stream of noise. **b)** The effect of attentional breadth on pupil size. The *y*-axis represents baseline-corrected pupil size in millimeters. The *x*-axis represents time in milliseconds since cue onset. The panels below the *x*-axis represent the order of the events in the experiments. Differently colored lines represent the three cue eccentricities (near, medium, far) corresponding to different levels of attentional breadth. The pink-shaded area represents the time window (2,500–2,600 ms) where the effect was the strongest, as determined through a cross-validation procedure. Error bands indicate the grand standard error. **c)** Mean baseline-corrected pupil size in millimeters as a function of cue eccentricity within the selected time window (2,500–2,600 ms). Error bars indicate the grand standard error. **d)** Attentional-breadth effect sizes for individual participants, sorted by effect size. (Color figure online)

Taken together, several studies have provided preliminary evidence for the effect of attentional breadth on pupil size; however, none of these studies simultaneously controlled for all potential confounds. Therefore, first of all, in the current study, we tested the same prediction while carefully controlling for all potential confounds. We believe that this is crucial because of the theoretical importance of the relationship between attentional breadth and pupil size. Second, we explored different operationalizations of attentional breadth, which in previous studies have not been clearly dissociated. Specifically, we distinguish three theoretically possible forms of attention: (1) the attentional spotlight can grow or shrink in the shape of a filled circle centered on the point of fixation, such that attention is never disengaged from central vision; in this view, increased attentional breadth is similar to a general increase in vigilance/attentiveness for anything that may appear at any location (Fig. 1a); (2) the attentional spotlight can grow or shrink in the shape of an annulus; in this view, increased attentional breadth is also similar to a general increase in vigilance, but with the added assumption that we either attend to (a specific eccentricity in) peripheral vision, or to central vision, but not to both (Fig. 1b); or (3) the attentional spotlight can be directed towards a single location in central or peripheral vision; this kind of attention is similar to spatial attention as manipulated in traditional cueing paradigms, and in this view increased attentional breadth is similar to a spatial shift of attention towards a location in the periphery (Fig. 1c). Each of these forms of attention allows for an increased or decreased emphasis on peripheral vision, but in different ways.

Taken together, in the present manuscript, we examine the effect of attentional breadth on pupil size while controlling for confounds that were not controlled for in previous research, and while exploring different operationalizations of attentional breadth. We further provide a crucial theoretical link between the effect of attentional breadth on pupil size on the one hand, and the AGT on the other hand.

## Materials and methods

### General

We conducted three experiments in order to test whether pupil size increases with increased attentional breadth. Each experiment’s methods, hypotheses, data sampling, and analysis plans were preregistered on the Open Science Framework. All data, analysis scripts, and supplementary materials are available online (https://osf.io/4nrgb/). The experiments were approved by the Ethics review board of the Department of Psychology at the University of Groningen (study approval code: PSY-2122-S-0139).

### Participants

In total, 111 (*N*_exp1_ = 30; *N*_exp2_ = 32; *N*_exp3_ = 49) first-year psychology students from the University of Groningen gave informed consent to participate in the study for course credits. For each experiment, we determined a target sample size and data exclusion criteria beforehand; we aimed for a target sample size of 30 participants for Experiment 1, and the same sample size was estimated for Experiment 2 based on a bootstrap power analysis conducted on the data from the first experiment (power = .81). Since we wanted to investigate an interaction effect in Experiment 3, and since we did not have a reliable effect-size estimate for a power analysis, we aimed for a target sample size of 40 participants. Prerequisites for retention of participant’s data were (1) full completion of the experiment, (2) efficacy of the staircase procedure (see Data Exclusion). All participants had normal or corrected-to-normal vision. All participants were unique for each experiment.

### Apparatus and data acquisition

The experiments were programmed in OpenSesame (Mathôt et al., 2012) using PyGaze for eye tracking (Dalmaijer et al., 2014). Stimuli were presented on a 27-inch monitor (1,920 × 1,080 pixels resolution; refresh rate: 60 Hz) and an EyeLink 1000 (sampling frequency of 1000 Hz; SR Research Ltd., Mississauga, Ontario, Canada), was used for eye tracking. Participants’ right eyes were recorded. All experiments were conducted in a dimly lit room.

### Procedure

Prior to the start of the experiment, the participant was well seated at about 60-cm distance from the computer monitor, with his or her chin placed on a chin-rest to keep the head in a stable position. First, a calibration-validation procedure was run. Before each trial, 1-point eye-tracker recalibration (“drift-correction”) was performed.

Throughout the experiments, participants were instructed to maintain their gaze in the center of the display, marked by a fixation dot. Each trial started with a centrally presented cue for 1,000 ms that indicated where a target was most likely to appear. The cue display was followed by the dynamic noise display that consisted of concentric annuli that differed in their respective radii, occupying different eccentricities: near (1.16°), medium (3.47°), and far (10.40°). The annuli were filled with dynamic, oriented noise patches that changed every 30 Hz. The spatial frequency of the noise patches decreased with eccentricity,

following the formula for cortical magnification as determined by Carrasco and Frieder (1997)

for the upper visual field. At a random moment^1^ within the last second of the dynamic noise presentation, the target (a subtle luminance increment or decrement) was briefly flashed for 30 ms, after which the dynamic noise continued for another 300 ms. On a majority of trials (80%) the target appeared at the cued annulus or location (valid trials) while on the remaining 20% it appeared at a noncued annulus or location (invalid trials). Participants’ task was to report whether the target was a luminance increment or decrement by pressing the left or right arrow on the keyboard, respectively. The sole difference between Experiment 1 and 2 was the type of the cue that was used, indicating the type of attentional breadth (Size or Location Condition). Specifically, in Experiment 1 we cued only the annulus (near, medium, or far) where the target was most likely to appear. To do so, we presented participants with symbolic cues (□, ○, ◁) that were indicative of each annulus. The mapping between cue symbol and eccentricity was counterbalanced across participants to ensure completely identical visual input in all conditions across participants. However, in Experiment 2, we cued both the annulus (near, medium, or far) and the location within the cued annulus (upper, lower, left, or right). Here, the cues were presented as a combination of codes for annuli (1/2/3 for near/medium/far) and codes for locations (U/D/R/L for up/down/right/left): for example, if U2 was presented, that meant that the target was most likely to appear at the upper location of the middle annulus. This mapping was constant across all participants, because otherwise the task would be too complicated. Experiment 3 combined both cueing approaches (hence, both attentional breadth forms) in a blocked design, keeping the same cues as in Experiment 2 for the Location Condition, where a specific location within a specific annulus was cued (Location Condition), and using only numeric cues (1, 2, 3) for the condition where only an annulus was cued (Size Condition). All cues were the same size (.69°) and colored in black. All stimuli were superimposed on a gray background.

To ensure that the accuracy on validly cued trials was maintained at approximately 70%, we implemented a 2-up-1-down (Exp. 1) or 3-up-1-down (Exps. 2 and 3) staircase procedure (Leek, 2001), separately for each eccentricity, varying the opacity of the target with 1% steps. In case three correct responses were given in a row, the target opacity would be decreased by 1%, thus increasing task difficulty, while in case of a single incorrect response, the target opacity would be increased by 1%, thus decreasing task difficulty. Throughout the experiment, participants received feedback in the form of a black checkmark (.92°) or X mark (.92°), indicating correct and incorrect responses, respectively.

All experiments consisted of 330 trials that were shuffled and divided into 10 blocks.

Experiment 1 and 2 consisted of 90 practice trials and 240 experimental trials. In Experiment 1, during practice trials, participants were shown both the cue in symbolic (□, ○, ◁) and written (“near,” “medium,” “far”) form to make sure that they learned the mapping; between practice blocks, participants were asked to verbally repeat to the experimenter which symbol corresponds to which annulus, in order to verify that participants remembered the cue–annulus mapping. Experiment 3 consisted of 30 practice trials and 300 experimental trials, half of which represented Location Condition (five successive blocks) and the other half represented Size Condition. The order of blocks was counterbalanced across participants.

### Data exclusion

Before analyzing the data, we checked if the staircase procedure was effective in keeping task difficulty equal across eccentricities. To that aim, after collecting the target sample size for each experiment, we compared participants’ performance when different eccentricities were cued by conducting a Bayesian repeated-measures ANOVA (with default parameters of JASP; JASP Team, 2022) only on validly cued trials, with Accuracy as a dependent variable and Cue Eccentricity as an independent variable. We used a Bayes factor (BF_01_ > 3 as a threshold for finding substantial evidence in favor of the null hypothesis. This condition was not met in Experiment 3 (initial BF_01_ = 2.25), so we determined for each participant separately absolute performance deviance (this step was also preregistered)—that is, how much performance differed across eccentricities based on the following formula:

|Accuracy(near)-Accuracy(overall)| + |Accuracy(medium)-Accuracy(overall)| +|Accuracy(near)-Accuracy(overall)|. Next, we iteratively excluded nine participants with the largest absolute performance deviance until we found substantial evidence for the null hypothesis. Finally, we recruited additional participants to reach again the target sample size, after which we ended up with BF_01_ = 3.63. In Experiment 2, the staircase procedure did not work for one participant (*M*_accuracy_ = .44) while one other participant made a large number of eye movements (*GazeError*_*max*_ = 19.07°). After these participants were replaced, we ended up with BF_01_ = 3.15. This condition was immediately met in Experiment 1 (BF_01_ = 6.29). Overall, the data from 100 participants was further analyzed (*N*_exp1_ = 30; *N*_exp2_ = 30; *N*_exp3_ = 40).

### Pupillary data: Preprocessing

Following the workflow for preprocessing pupillary data that we described elsewhere (Mathôt & Vilotijević, 2022), we first interpolated blinks and downsampled the data by a factor of 10. Also, we converted pupil size measurements from arbitrary units to millimeters of diameter by using the formula specific to our lab (Wilschut & Mathôt, 2022). Next, we baseline-corrected the data by subtracting the mean pupil size during the first 50 ms after the onset of the cue (baseline period) from all subsequent pupil-size measurements on a trial-by-trial basis. Trials containing baseline pupil sizes of ±2 *z*-scores were considered outliers, and hence excluded from the data. Missing trials (due to technical issues with recording in Experiment 2 and 3) were excluded as well (0.05%). In total, 1,576 trials (Total = 5.91%; Exp. 1 = 5.58%, Exp. 2 = 5.44%, Exp. 3 *=* 6.51%) were excluded from the data.

### Reporting of results

We analyzed eye-tracking data from all 100 participants performing a visual discrimination task. As mentioned above, we conducted three separate experiments, which are described in detail above (see Procedure) and analyzed separately as described in the Supplemental Information; however, because the task and design of all three experiments were very similar, and we wanted to test the interaction with the type of attentional breadth, we will focus on the combined data below.

## Results

### The effect of attentional breadth on pupil size

To investigate the effect of attentional breadth on pupil size, we ran a four-fold cross-validation analysis on a predetermined time-window of 750-3000 ms after the onset of the cue to assess differences in pupil size across eccentricities, after which a single linear mixed effects (LME) is conducted for the full dataset (this procedure is described in more detail in Mathôt & Vilotijević, 2022).

Our model included pupil size as dependent variable, Cue Eccentricity as fixed effect (coded ordinally: −1 = *near*, 0 = *medium*, 1 = *far*; this was the case for subsequent analyses as well), and by-participant random intercepts and slopes. The lower boundary of our period of interest (750 ms) was decided on the basis of the finding that a voluntary shift of covert visual attention towards a bright or dark surface affected pupil size from about 750 ms after cue onset (Mathôt et al., 2013). The upper boundary (3000 ms) was set to the first moment at which the target could appear in any of the three experiments. The effect of Cue Eccentricity on pupil size emerged around 1,750 ms after cue onset and the cross-validation analysis showed that it reached maximum around 2,500 and 2,600 ms after cue onset (*t* = 2.75, *p* = .006). Specifically, and in line with our hypothesis, we found that the pupil size increased with increasing attentional breadth (see Fig. 2b–c).

Next, to test whether the effect of attentional breadth differed between the Size Condition and Location Condition, we focused on mean pupil size during the 2,500–2,600-ms window as selected by the cross-validation analysis. We conducted an LME analysis with mean pupil size during the selected interval as dependent variable, Cue Eccentricity and Attentional Breadth Type (reference: Size Condition) as fixed effects, and by-participant random intercepts (a model with by-participant random slopes failed to converge). This revealed, as before, a main effect of Cue Eccentricity (*b* = 0.008, *SE* = 0.003, *t* = 2.77, *p* = .006), but no main effect of Attentional Breadth Type (*b* = 0.008, *SE* = 0.005, *t* = 1.58, *p* = .115), and—more importantly—no interaction between Cue Eccentricity and Attentional Breadth Type (*b* = −0.003, *SE* = 0.004, *t* = 0.74, *p* = .457).

Finally, we visualized the magnitude of individual differences across participants in the strength of the effect of attentional breadth on pupil size (Fig. 2d); here, the “Attentional Breadth” effect corresponds to the Pearson correlation coefficient between Cue Eccentricity and mean pupil size during the 2,500–2,600 ms window, determined for each participant separately. About two-thirds of the participants showed an effect in the hypothesized direction; in other words, although the effect of attentional breadth is highly reliable at the group level, there is a lot of interindividual variability.

Taken together, we found an effect of attentional breadth on pupil size, but this effect did not reliably depend on the type of attentional breadth (Size Condition vs. Location Condition).

### Behavioral cueing effect

**Fig. 3:**
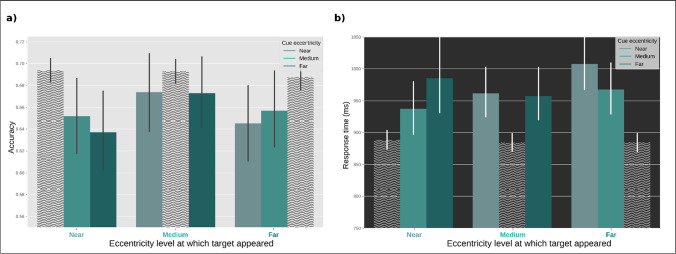
Behavioral results. **a)** Accuracy as a function of the eccentricity at which the target appeared (*x*-axis) and the eccentricity that was cued (bars). **b)** Reaction times as a function of the eccentricity at which the target appeared (*x*-axis) and the eccentricity that was cued (bars). Shaded wavy bars represent valid conditions. Error bars represent the grand standard error. (Color figure online)

Next, to test the behavioral cueing effect, we ran Linear Mixed Models (LMM) testing a model that included Accuracy as dependent variable, Cue Validity as fixed effect, and by-participant random intercepts and slopes for Cue Validity. The results showed a behavioral cueing effect; that is, participants were more accurate on valid versus invalid trials (*b* = 0.17, SE = 0.03, *z* = 5.02, *p* < .001; Fig. 3a).

### Task difficulty

As explained in the Methods section, the accuracy across conditions was controlled by a staircase procedure, keeping it at around 70% for validly cued trials, to rule out the potential effect of mental effort. However, participants may have still perceived some conditions to be more difficult than others, and consequently responded more slowly to those trials. To check for this, we ran a Linear Mixed Model (LMM) testing a model that included Reaction Times as dependent variable, Cue Eccentricity as fixed effect, and by-participant random intercepts and slopes for Cue Eccentricity. The results showed that there were no significant differences in RT across conditions (*b* = −2.68, *SE* = 4.20, *z* = −.64, *p* = .523; Fig. 3b). In other words, there was no difference in task difficulty between different levels of attentional breadth, neither in terms of accuracy nor reaction times.

## Discussion

We report three experiments that investigated whether pupil size increases with increasing attentional breadth, and if this effect depends on the type of attentional breadth, that is, whether attentional breadth is induced through changes in the *size* (see Fig. 1a–b) or the *location* of the attentional spotlight (see Fig. 1c). Large pupils are known to benefit detection of faint stimuli in peripheral vision (Mathôt, 2020; C.-A. Wang et al., 2021; Woodhouse, 1975); therefore, the question of whether pupils reflexively dilate in response to increased attentional breadth is crucial for a better understanding of pupil responses as a form of *sensory tuning*: a subtle adjustment of the eyes to optimize visual-information intake for a given situation (Mathôt, 2020; see also Franke et al., 2022).

We found that pupil size increases with increasing attentional breadth. As shown in Fig. 2b, the pupil slightly dilates during the cue presentation (1,000 ms) after which it rapidly constricts once dynamic noisy annuli are shown; this constriction reflects the typical pupillary response to visual stimulation and thus is consistent across conditions. Next and most importantly, pupil traces begin to differ between conditions around 1,750 ms after the cue onset, reaching a maximum difference around 2,500–2,600 ms, and lingering until the target’s presentation. This means that when the target was expected to appear at the near level of attentional breadth (encompassing central vision), pupils were smaller than when the target was expected at the far or medium levels of attentional breadth (encompassing peripheral vision). Additionally, our data demonstrate that this effect does not, or hardly, depend on the type of attentional breadth, that is, on whether participants attend to entire circles at different eccentricities (Size Condition) or to specific locations at different eccentricities (Location Condition).

Although this was not the primary focus of our study, our behavioral results also speak to the question of whether, when an entire circle is cued (our Size Condition), attention takes the form of a filled circle (Fig. 1a) or of a “doughnut”/annulus (Fig. 1b). That is, are participants able to attend exclusively to the cued circle without also attending to the smaller circles within the cued circle? The answer appears to be yes: we found that attention was *selective* for the cued circle (Nobre & Kastner, 2014), as participants were consistently better performing on validly cued trials across *all* eccentricities (Fig. 3). This suggests that attention was deployed in the shape of differently sized annuli, as depicted in Fig. 1b, rather than in the shape of differently sized spotlights/filled circles, as depicted in Fig. 1a. This is noteworthy because it implies an important difference between the concepts of attentional breadth and zoom-lens theory: The latter assumes attentional deployment in a form of differently sized filled circles (this also matches the concept of “attentional scaling” proposed by Lawrence et al., 2020), whereas attentional breadth assumes that attention is deployed in the form of differently sized annuli (Jefferies & Di Lollo, 2015; this also matches the concept of “doughnut” attention; Muller & Hubner, 2002). Our results suggest that attention can be distributed in the shape of a doughnut.

Finally but importantly, our results add to the AGT by offering a functional explanation for *why* there is a correlation between pupil size and modes of behavior (Gilzenrat et al., 2010; Jepma & Nieuwenhuis, 2011; van den Brink et al., 2016). Specifically, an exploration mode of behavior is likely characterized by an emphasis on peripheral vision, and through this route may lead to pupil dilation. The same logic applies to other situations that are associated with pupil dilation. For example, high arousal is associated with pupil dilation (and thus high LC tonic activity mode; Binda & Murray, 2015; Bradley et al., 2008); thus far, arousal-related pupil dilation has been interpreted as an epiphenomenal physiological response. However, we propose that arousal-related pupil dilation is a way in which the visual system tunes itself to best serve the demands of the situation; specifically, in a situation that elicits high arousal (e.g., when you are afraid) it is crucial to be able to detect things anywhere in the visual field with little concern for their details; thus, the visual system recognizes that detection is more important than discrimination, and pupils consequently dilate to provide greater sensitivity.

The rationale above presupposes that the link between attentional breadth and pupil size confers a behavioral advantage that the visual system makes use of. The design of the present study is not well-suited to demonstrate such a behavioral advantage for two main reasons: First, we used an interleaved staircase that kept performance constant across conditions and time; this was required to rule out task difficulty as a confound, yet also artificially reduced correlations between performance and pupil size. Second, we did not induce changes in pupil size in the current paradigm, and although it is possible to look at correlations between spontaneous fluctuations in pupil size and behavior, it is difficult if not impossible to draw causal inferences based on such data. Therefore, to establish a behavioral advantage of the link between attentional breadth and pupil size, future studies could use paradigms in which task difficulty is not, or not continuously, adjusted through a staircase procedure, and in which pupil size is experimentally induced (Mathôt et al., 2023). Studies of this general kind will be a crucial next step to understand the function of cognitively driven changes in pupil size.

In sum, we demonstrate that pupil size increases with covert shifts of attention to the peripheral visual field, that is, with increasing attentional breadth. We propose that, more generally, cognitively driven pupil dilation reflects, among other things, an emphasis on peripheral vision over foveal vision, and that this may explain why the pupil dilates in response to increased arousal or a switch to an exploration mode of behavior as postulated by the AGT.

### Supplementary Information

ESM 1 (DOCX 19 kb)

## Footnotes

1 The exact time window in which the target appeared was 2,000–3,000 ms in Experiment 1 and 2,500–3,500 ms in Experiments 2 and 3. However, this did not make any difference, as the latency of the attentional breadth effect was the same in all three experiments (~ 2 s since the cue onset; see Fig. 2b).
