# Supplementary material for: Emphasis on peripheral vision is accompanied by pupil dilation
Source: Psychon Bull Rev. 2023 Apr 17;30(5):1848–56. doi: 10.3758/s13423-023-02283-5 (PMC10716087; doi:10.3758/s13423-023-02283-5)
Supplement: Supplementary file 1 — (DOCX 19 kb) [file 13423_2023_2283_MOESM1_ESM.docx]

**Supplemental information**

To test the attentional breadth effect in each experiment separately, we run LME with Mean Pupil Size during the selected time window (2500-2600 ms), determined by cross-validation analysis run on the collapsed data, as a dependent measure while Cue Eccentricity (ordinal: -1 = *near*, 0 = *medium*, 1 = *far*) was a fixed effect and by-participant random intercepts and slopes. Additionally, we tested cueing effect by conducting LMM using Accuracy as the dependent variable while Cue Validity was included as a fixed effect. The random-effect structure included a by-subjects random intercept and random slopes for Cue Validity.

*Experiment 1 - Size Condition.* The main effect of Cue Eccentricity was significant, *b* = 0.012, *SE* = 0.005, *t* = 2.696, *p* = 0.007. Looking at the behavioral performance, we observed a cueing effect, suggesting that participants were more accurate on valid trials, (*b* = 0.14, *SE* = 0.05, *z* = 2.61, *p* = 0.009).

*Experiment 2 - Location Condition.* When a specific location within a certain annulus was cued the pupillary effect did not reach significance (*b* = 0.004, *SE* = 0.004, *t* = 1.05, *p* = 0.315), although the pattern of results was still in line with overall results. The cueing effect was now even more prominent, *b* = 0.25, *SE* = 0.05, *z* = 4.63, *p* = 0.000.

*Experiment 3 - Size and Location Conditions.* The pupillary results were again in the hypothesized order, however, the results did not reach overall significance (*b* = 0.004, *SE* = 0.004, *t* = 1.21, *p* = 0.262). When analyzing conditions─Size Condition and Location Condition─separately, the pupillary results were in the hypothesized order, however, none of them reached significance: Size Condition: *b* = 0.001, *SE* = 0.005, *t* = 0.23, *p* = 0.821; Location Condition: *b* = 0.007, *SE* = 0.005, *t* = 1.45, *p* = 0.147. As regards the cueing effect, we observed the overall cueing effect in Experiment 3, *b* = 0.20, *SE* = 0.05, *z* = 4.31, *p* = 0.000). This was also true when only Location Condition was tested (*b* = 0.23, *SE* = 0.07, *z* = 3.42, *p* = 0.001) as well as for Size Condition (*b* = 0.18, *SE* = 0.07, *z* = 2.67, *p* = 0.008).

**[INSERT FIGURE 4 HERE]**

Figure 4. *a)* Mean baseline-corrected pupil size expressed in millimeters as a function of cue eccentricity across experiments. *b)*. Accuracy as a function of cue validity across experiments and corresponding conditions. Error bars indicate 95% confidence interval.
